# Solitary Fibrous Tumor: Integration of Clinical, Morphologic, Immunohistochemical and Molecular Findings in Risk Stratification and Classification May Better Predict Patient outcome

**DOI:** 10.3390/ijms22179423

**Published:** 2021-08-30

**Authors:** Isidro Machado, María Gema Nieto Morales, Julia Cruz, Javier Lavernia, Francisco Giner, Samuel Navarro, Antonio Ferrandez, Antonio Llombart-Bosch

**Affiliations:** 1 Pathology Department, Instituto Valenciano de Oncología, 46009 Valencia, Spain; jcruz@fivo.org; 2 Patologika Laboratory, Pathology Department, Hospital Quiron-Salud, 46009 Valencia, Spain; 3 Zoology Department, University of Valencia, 46009 Valencia, Spain; m.gema.nieto@uv.es; 4 Department of Oncology, Instituto Valenciano de Oncología, 46009 Valencia, Spain; javilavernia@gmail.com; 5 Pathology Department, University Hospital “La Fe”, 46009 Valencia, Spain; francescg@hotmail.com; 6 Pathology Department, University of Valencia, 46009 Valencia, Spain; samuel.navarro@uv.es (S.N.); Antonio.ferrandez@uv.es (A.F.); antonio.llombart@uv.es (A.L.-B.)

**Keywords:** solitary fibrous tumor, risk stratification systems, STAT6, APAF-1, *HTER* mutation, *p53* mutation

## Abstract

Although solitary fibrous tumors (SFTs) have an unpredictable evolution, some specific clinicopathologic factors have been associated with the final outcome. We retrieved clinical, pathological and molecular data of 97 patients with a histological diagnosis of SFT and Signal transducer and activator of transcription 6 (STAT6) positivity. We retrospectively studied the pathological factors predictive of recurrence/metastasis and compared them with the clinical outcome. A wide immunohistochemical study and molecular analysis to detect *NAB2/STAT6* gene fusion, tumor protein-53 (*TP53)* and/or (telomerase reverse transcriptase) *TERT* promotor mutation were performed. The risk of metastasis was calculated using the Demicco risk stratification system (RSS). The results were combined and examined to assess the accuracy of risk stratification and classification. The most common location was in non-extremities; 66% were located in soft tissue or subcutaneous areas and 92.8% in deep locations. On microscopic analysis, 38.1% of tumors revealed hypercellularity with a predominant patternless and/or hemangiopericytic growth pattern; 13.4% had ≥4 mitoses/10HPF; 16.5% showed necrosis, and almost half the tumors showed at least focal myxoid areas. Dedifferentiation was observed in three tumors. Immunomarker expression in SFTs was as follows: CD34 92.9%, CD99 57.1%, Bcl2 67.9%, neuroendocrine markers (at least 1) 25.7%, Desmin 14.3%, CK(AE1/AE3) 3%, Apoptotic Protease Activating Factor (APAF-1) 87% and finally Ki-67 ≥ 10% in 14.4%. The *NAB2/STAT6* gene fusion was detected in 50 tumors. After a median follow-up of 90 months, 9.3% recurred, 11.3% metastasized, 10.3% died of disease and 76.2% were free of disease. *TERT* mutations were detected in 40.6% of the SFTs; the *TP53* mutation was detected in 17%, and only 9.3% showed both mutations. According to the Demicco RSS, 6.1%, 11.3% and 82.4% of the tumors were classified as high, intermediate or low-risk of metastasis, respectively. All high-risk tumors had ≥4 mitoses/10HPF, necrosis, Ki-67 ≥ 10, *HTER* and/or *TP53* mutation and poor evolution. The intermediate risk SFTs with worse evolution displayed the *HTER* mutation. Almost all low-risk tumors had a favorable evolution, although four showed at least one adverse factor (Ki-67 ≥ 10, ≥4 mitoses/10HPF or high tumor size) and had a worse evolution. An integration of clinical, morphologic, immunohistochemical and molecular findings may improve risk stratification and classification and better predict patient outcome. The unfavorable course seems to be more frequent in high-risk SFTs, although it is not exceptional in low-risk SFTs either; hence, a long-term follow-up is required independently of the assigned risk stratification score. The inclusion of molecular findings in risk stratification systems could improve the precision in the classification of SFTs, especially those of intermediate risk. Future studies will be required to determine the most effective way to incorporate molecular analyses into RSS on SFTs. The coexistence of several adverse factors such as ≥4 mitoses/10HPF, necrosis, Ki-67 ≥ 10%, mutations in *HTER* and/or *p53* may suggest a closer clinical follow-up regardless of the histological appearance of the tumor.

## 1. Introduction

Solitary fibrous tumors (SFTs) are mesenchymal neoplasms that can occur at any location, especially pleural, meningeal or extrapleural sites. Location in limbs is infrequent [1,2,3,4,5,6,7,8,9,10,11,12,13,14,15,16,17,18,19,20,21,22,23,24,25,26,27,28,29,30,31,32,33,34,35,36,37,38,39,40].

The evolution of SFTs is often uncertain [2,3,4,5,6,7,8,9,10]. Although most cases evolve favorably, a small group can progress towards dissemination, generally pulmonary, as well as other locations, and will lead to patient death in the absence of effective treatments [1,2,3,4,5,6,7,8,9,10,11,12,13,14,15,16,17,18,19]. Thus, valid and reproducible tools should be developed in order to design risk-tailored treatment and risk-adjusted follow-up.

A patternless and hemangiopericytic growth with variably fibrosis and collagenous deposits are characteristic, but SFTs can show many faces, including round cells, giant-cells, myxoid areas, pleomorphic pattern, fat-forming tumors and dedifferentiated forms [1,2,3,4,5,15,16,17,18,19,20,21,22,23,24,25].

Strong and diffuse nuclear immunoreactivity for Signal transducer and activator of transcription 6 (STAT6) is almost always diagnosed in SFTs, and CD34, Bcl-2 and CD99 immunoexpression is common [1,2,3,4,5,6,7,8,9,10,11,12,13,14,15,16,17,18,19,20,21,22,23,24,25]. Aberrant epithelial, muscular or neuroendocrine marker expression has been described which may lead to confusion with other tumors that share a similar morphology [15,16,17,18,19,20,21,22,23,24,25,36]. The fusion gene *NAB2/STAT6* and its variants confirm a diagnosis of SFT in those cases with unconvincing STAT6 immunoreactivity, and specific gene fusions have been related to prognosis and tumor location [6,7,8,9,10,11,12,13,14,26,27,28,29,30,31,32,33,34,35,36,37,38,39,40]. STAT6 nuclear expression is not exclusive to SFTs and has also been reported in dedifferentiated liposarcoma, glioma-associated oncogene homolog 1 (*GLI1)*-amplified tumor, Kaposi sarcoma and Hodgkin as well non-Hodgkin lymphomas [6,12,13,14,15,16,17,18,19,20,21,22,23,24,25,36,37,38,39,40].

Several risk-stratification systems (RSS) have been described [1,2,3,4,5], although the Demicco et al. RSS seems to be the most widely implemented [1,2,14]. While most SFTs categorized as low-risk by the Demicco system follow an apparently benign course, some cases may have late relapse or metastases; hence, the RSS is not perfectly specific in predicting the evolution in all cases [1,2,14]. In addition, molecular results have not so far been included in any of the current RSS [1,2,14]. Recently, loss of Apoptotic Protease Activating Factor (APAF-1) expression has been associated with poor prognosis in SFTs, but these findings need to be confirmed in larger series [41].

We have previously published a small series of SFTs with at least one histological factor associated with aggressive behavior and explored the correlation between the adverse histological findings and molecular profile with tumor behavior [42]. The main goal of the present study was to confirm our previous results including additional tumors, mainly SFTs categorized as low-risk by Demicco et al. [1,2] and correlate the clinical, histological and immunohistochemical findings, *TP53* mutational status and *TERT* promotor mutational status with clinical outcome.

## 2. Results

### 2.1. Clinicopathological and Histopathological Findings

**Figure 1:**
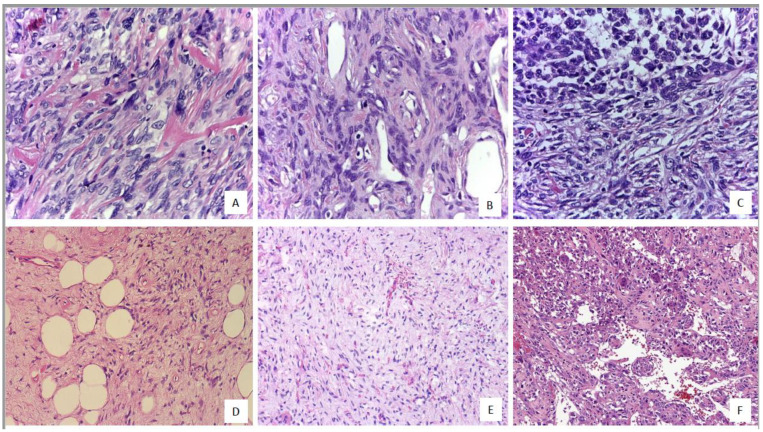
(**A**) Soft tissue solitary fibrous tumor (SFT) with spindle/ovoid growth pattern and fibrocollagenous tissue, hematoxylin and eosin (H&E) 40×. (**B**) SFT with collagenous stromal tissue and focal hemangiopericytoma-like pattern, H&E 40×. (**C**) Dedifferentiated SFT with abrupt transition between a well-differentiated area with a patternless appearance and dedifferentiated zone with round cell sarcoma appearance, H&E 40×. (**D**) A lipomatous/fat-forming SFT with mature adipocytes intermixed with spindle-ovoid cells, H&E 20×. (**E**) Myxoid SFT, H&E 20×. (**F**) SFT with multinucleated/giant stromal cell, H&E 20×.

The clinicopathological and follow-up data are shown in Table 1 and Table 2. The histologic findings are shown in Figure 1 and summarized in Table 3. The median follow-up was 90 months. In the microscopic analysis, 38.1% of tumors revealed hypercellularity with a predominant patternless and/or hemangiopericytic growth pattern (Figure 1); 13.4% had ≥4 mitoses/10HPF; 16.5% showed necrosis and myxoid areas were frequent (Figure 1). Dedifferentiation was observed in three tumors (Figure 1). Rare histological patterns were found in sporadic cases (Figure 1).

### 2.2. Immunohistochemistry Results

**Figure 2:**
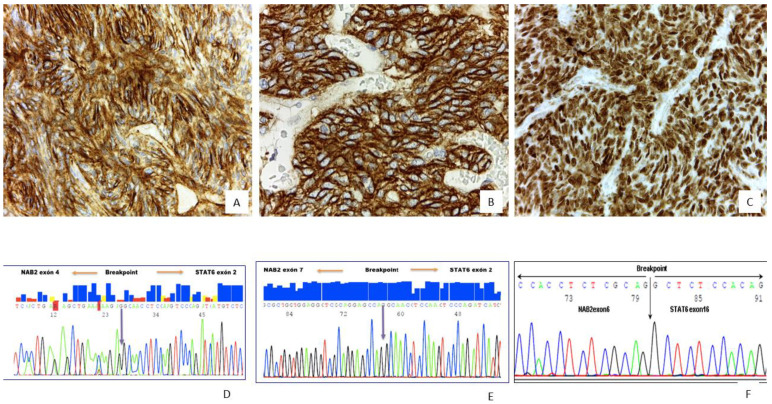
(**A**) Diffuse and strong CD34 positivity in tumor cells in SFT, 40×. (**B**) Diffuse (membranous) CD99 immunoreactivity in tumor cells in SFT, 40×. (**C**) Diffuse and strong nuclear STAT6 positivity in tumor cells of SFT, 40×. (**D**–**F**) Molecular analysis in three different SFT cases with *NAB2/STAT6* gene fusion; the breakpoints were *NAB2 exon 4-STAT6 exon 2*, *NAB2 exon 7-STAT6 exon 2* and *NAB2 exon 6-STAT6 exon 16)* respectively.

The representative status of the immunohistochemical profile is shown in (Figure 2) and summarized in Table 4. All tumors were diffusely positive for STAT6 (Figure 2C), CD34, CD99 (Figure 2A,B) and Bcl2 which were frequent, and neuroendocrine markers (at least 1), Desmin, CK(AE1/AE3) were detected sporadically. APAF-1 immunoexpression was detected in 87% of the SFTs, and Ki-67 ≥ 10 was found in 14.4% of the tumors.

### 2.3. Molecular Study

**Figure 3:**
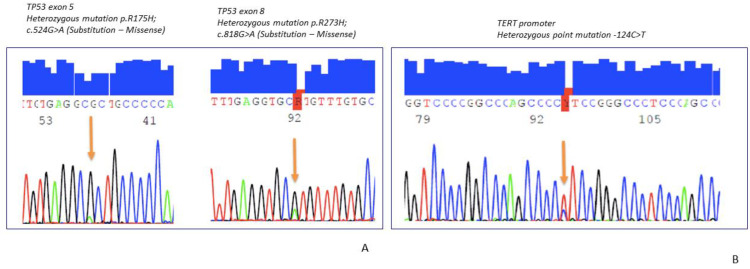
(**A**) High-risk SFT with *TP53* mutation, (**B**) Intermediate-risk SFT with *HTER* mutations.

**Figure 4:**
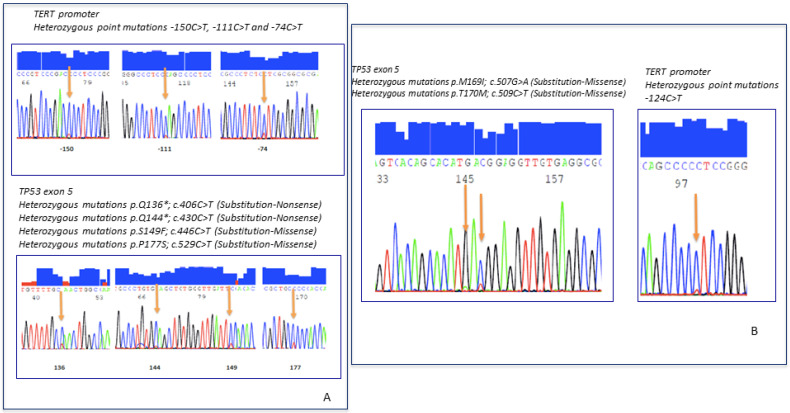
(**A**) Molecular analysis in high-risk SFT shows TP53 and TERT promoter mutations. (**B**) Dedifferentiated SFT with TP53 and TERT promoter mutations.

Molecular results are summarized in Table 5. *NAB2*-exon 4/*STAT6*- exon 2 gene fusion was detected in 35 cases, *NAB2*-exon 6/*STAT6*-exon 16/17 in 15 cases (Figure 2D–F). *TP53* and *TERT* promotor status is summarized in Table 5. *TERT* mutations or *TP53* mutations were detected in 40.6% and 17% of the tumors (Figure 3A,B), respectively. A total of 9.3% of the SFTs showed both mutations (Figure 4A,B).

### 2.4. Risk of Recurrence/Metastasis

The risk stratification system classifications are described in Table 6. Regarding molecular analysis, non-informative results for RNA (*NAB2/STAT6* fusion gene) were given in 2 high-risk SFTs, 5 intermediate-risk SFTs and 40 low-risk SFTs. All these cases showed strong and diffuse nuclear STAT6 immunoreactivity. Eleven cases were non-informative for *HTER* and/or *p53* molecular status; these cases were all classified as low-risk of metastasis.

The correlation between clinical and histological findings (mitosis and/or necrosis), the Ki-67 index and molecular status with clinical outcome (metastasis and/or died of disease versus free of disease) in high-risk and intermediate-risk tumors are described in Table 7 and Table 8. The correlation of clinical and histological findings (mitosis and/or necrosis), the Ki-67 index and molecular status with clinical outcome in low-risk tumors with worse evolution is described in Table 9. We did not find any correlation between APAF-1 status and clinical outcome.

## 3. Discussion

Strong and diffuse nuclear immunoreactivity for STAT6 is a very useful tool for the diagnosis of SFTs, although unexpected epithelial, muscular or neuroendocrine marker expression has also been described in these tumors and may lead to confusion with other neoplasms with hemangiopericytic growth patterns [2,15,16,17,18,19,20,21,22,23,24,25,33,42]. STAT6 expression has also been reported in dedifferentiated liposarcoma and *GLI1*-amplified tumors; hence, in cases with overlapping morphology and STAT6 immunoreactivity, additional molecular studies are needed to establish a definitive diagnosis [2,15,16,17,18,19,20,21,22,23,24,25,33,34,35,36,37,38,39,40,41,42]. Detection of the specific fusion gene *NAB2/STAT6* and its variants confirm a diagnosis of SFT, especially in rare clinical settings, unusual histological findings or unexpected immunohistochemical results. Moreover, specific gene fusions have been related to prognosis and tumor location [7,8,11,12,26,27,28,29,30,31,32,35,36,37,38,39,40,41,42]. Various risk-stratification systems (RSS) have been described, with the Demicco et al. system being the most widely implemented, as recommended in the World Health Organization (WHO) blue book [1,2,3,4,5,14].

Most low risk SFTs categorized by Demicco et al. and other RSS follow an apparently indolent course. However, some of these cases may have late recurrence/relapse and/or metastases leading to uncertainty and skepticism among oncologists regarding the specificity of RSS in correctly predicting evolution in all cases [1,2,3,4,5,14]. Furthermore, molecular results have not been included in any of the present RSS so far [1,2,3,4,5,6,7,8,42].

Regarding histological predictive factors of aggressiveness, high mitotic counts with a general agreement of ≥4/10HPFs represent the strongest predictor of malignant behavior, as confirmed in the present series where all tumors categorized as high-risk and many of the tumors classified as intermediate-risk revealed ≥4/10HPFs [1,2,3,4,5,6,7,8,42]. Likewise, necrosis was present in the same group of tumors (all high-risk and many intermediate-risk).

Of all the immunohistochemical markers applied in this series, only Ki-67 ≥ 10 was associated with poor evolution. Similar to mitosis and necrosis, this finding was also found in all SFTs categorized as high-risk. Ki-67 has only been included in the Diebold et al. RSS [3], and although further confirmation is needed, this factor may represent an additional variable that could provide valuable predictive information on tumor evolution. In the present series, half the tumors categorized as low-risk by the Demicco et al. system but with worse evolution (late recurrences or metastasis) showed Ki-67 ≥ 10.

Recently, loss of APAF-1 immunoexpression has been associated with the progression and poor prognosis in SFTs [41]. APAF-1 inactivation may lead to impaired apoptotic function, and eventually may contribute toward malignant SFT transformation [41]. Nevertheless, this finding needs to be confirmed in larger series. We failed to find this association in the present study, despite some SFTs with aggressive evolution (dedifferentiated tumors) showing loss of APAF-1 expression by immunohistochemistry.

Molecular studies in SFTs have been progressively implemented in many centers for diagnosis and prognosis. SFTs with the most common canonical *NAB2 exon 4-STAT6 exon 2* fusion variant are often located in the thorax and are less cellular with abundant fibrosis [8,9,10,11,12,28,29,30,31,32,37,38,39,40]. These tumors have a higher tumor age, larger tumor size, lower mitotic activity and lower recurrence rate [28,29,30,31,32,37,38,39,40]. However, those tumors with *NAB2 exon 6-STAT6 exon 16/17* fusion variants typically show a more cellular round to ovoid cell morphology and are often located in the deep soft tissue of the intra-abdominal/retroperitoneal/pelvic region or in central nervous system [28,29,30,31,32,37,38,39,40]. SFTs with *NAB2 exon 6-STAT6 exon 16/17* fusion occur in a significantly younger age group, showing higher mitotic activity and a higher recurrence rate [39].

Recently, a new study reported that SFTs with the *NAB2 exon 4-STAT6 exon 2* fusion variant show a transcriptional signature enriched for genes involved in DNA binding, gene transcription and nuclear localization, whereas SFTs with the *NAB2 exon 6-STAT6 exon 16/17* fusion variants were enriched for genes involved in tyrosine kinase signaling, cell proliferation and cytoplasmic localization [40]. In addition, Georgies T et al. have reported prognostic significance in SFTs depending on *STAT6* domain composition [40]. They categorized SFTs as either *STAT6-TAD* (contained only the transactivation domain of STAT6) or *STAT-Full* (fusions with most of the STAT6 domain intact). Tumors with *STAT6-TAD* fusions had a higher mitotic count (*p* = 0.016) and were associated with poor prognosis [40].

We did not observe any direct association of gene fusion variants with aggressiveness or location of any histologic or phenotypic profile in the present series.

A limitation of the present study was the fact that 48.5% of the samples (47/97) showed non-informative RNA results for the detection of the *NAB2/STAT6* fusion gene. Nevertheless, all cases in the present series showed strong and diffuse STAT6 nuclear expression by immunohistochemistry. This finding of strong and diffuse nuclear STAT6 immunoreactivity has previously been implemented in many laboratories as a surrogate for the molecular analysis in solitary fibrous tumors considering the good correlation between the nuclear STAT6 overexpression and the presence of the *NAB2/STAT6* fusion gene [1,2,3,4,5,6,7,8,42]. Furthermore, since the *NAB2/STAT6* fusion gene represents an intrachromosomal inversion, a non-informative or negative result is not exceptional in gene fusion molecular studies.

Reverse transcription-polymerase chain reaction (RT-PCR) may not be the most suitable method to detect fusion genes in SFTs, and more sensitive techniques such as next generation sequencing (NGS) or RNAseq may have improved the quality of the results. However, these very advanced molecular ancillary tests are also more expensive and not widely available in all pathology labs. RNA analysis failure using RT-PCR could be explained by limited material in the case of a core biopsy or by fixation issues or tissue conservation [1,2,3,4,5,6,7,8,42]. Furthermore, although the present study used the most frequent primers for RT-PCR, they would not necessarily cover the full spectrum of possible gene fusion types in solitary fibrous tumors. Indeed, RNA analysis failure may be considered multifactorial.

*TERT* promoter mutations and the *TP53* mutation have been associated with malignant behavior in SFTs [7,8,12,35,36,37,38,39,40,41,42]. The overall prevalence of the *TP53* mutation in the current series was low, in contrast to the *TERT* mutation that was found in almost half the tumors. Many of the high-risk tumors had *TP53* mutations, *HTER* mutations or both [7,8,12,35,36,37,38,39,40,41,42]. Likewise, intermediate-risk SFTs with the *HTER* mutation in the present series showed poor evolution, in line with the previous observation of Demicco et al. where the *TERT* mutation probably provides no additional prognostic information on tumors already classified as low or high risk [7]. At present, despite the various studies confirming that the *HTER* promotor and/or *TP53* alterations in SFTs are associated with prognosis, no risk stratification system has so far incorporated these molecular factors [1,2,3,4,5,6,7,8]. In the present series, there were only 11 cases with non-informative results for *HTER* and *p53* molecular status, all of which were classified as low-risk of metastasis.

Four RSS have been proposed in the literature that classify SFTs into three (low, intermediate, high-risk) or two categories (low vs. high-risk) depending on the system [1,2,3,4,5,14]. The most commonly implemented is the Demicco et al. RSS which includes both clinical and histological variables [1,2,14]. The Diebold et al. system includes the Ki-67 index, which is not included in the other RSS [3]. Although presumably all dedifferentiated SFTs show other adverse clinical or histological factors, this histological characteristic has not been included in any of the RSS [1,2,3,4,5,14].

In the present series, all SFTs classified as high-risk by Demicco et al. had Ki-67 ≥ 10. We had four cases categorized as low-risk by Demicco et al. but with a worse clinical evolution. Two out these four cases had a Ki-67 index higher than 10 and when applying the Diebold et al. RSS [3], those cases were classified as high-risk tumors; hence, when the systems disagree on risk stratification, the clinical evolution becomes more difficult to predict, which may suggest an imperfect specificity in predicting accurate evolution in some RSS. Incorporating the Ki-67 index might provide prognostic information in some cases independently of the histologic appearance of the tumor. However, Ki-67 results in the present series need to be validated in independent SFT series.

The identification of dedifferentiation in SFTs is very important because new evidence has revealed that antiangiogenics are effective, and their use as a first line of treatment should be considered in SFTs, but not in dedifferentiated SFTs for which chemotherapy is more effective [8].

It would be interesting in the future to study the tumor/stromal tissue interaction, and whether the amount of collagen and/or the type of collagen fibers could have any prognostic significance in these tumors. At present, hypercellularity in solitary fibrous tumors, which usually correlates with a lower amount of collagen, has been correlated in some studies with a somewhat more aggressive evolution in SFTs, although hypercellularity is not necessarily always associated with aggressive evolution [1,2,3,4,5,14].

In conclusion, risk assessment still remains a challenging issue in SFT classification and the final outcome. However, the integration of clinical, morphologic, immunohistochemical and molecular findings may improve risk stratification and classification of SFTs and could guide the clinician when designing risk-adjusted treatment and follow-up. Regardless of the assigned risk stratification score, SFTs may require long-term follow-up considering that low-risk tumors may occasionally show a non-indolent evolution. The inclusion of molecular findings in RSS could improve precision in the classification of SFTs, especially those of intermediate risk. Nevertheless, future studies are required to determine the most effective way to incorporate molecular analyses into RSS on SFTs. The coexistence of several adverse factors such as ≥4 mitoses/10HPF, necrosis, Ki-67 ≥ 10%, mutations in *HTER* and/or *p53* may suggest the need for a closer clinical follow-up regardless of the histological appearance of the tumor.

## 4. Material and Methods

### 4.1. Patients and Samples

We collected 97 cases of histologically proven SFTs having strong and diffuse nuclear STAT6 positivity. Formalin-fixed, paraffin-embedded tissue (FFPET) was retrieved from the archives at the Pathology Department, Clinical Hospital, University of Valencia; Hospital Universitari i Politécnic La Fe and Instituto Valenciano de Oncología (IVO) Valencia. This study was conducted in accordance with the principles of the Declaration of Helsinki and approved by the local Ethics Committee (IVO 2018-28). Clinical data (age, gender, tumor site, tumor location, size, tumor depth, treatment) and follow-up data (recurrence, metastases and final outcome) were also retrieved.

### 4.2. Histopathology

All the available H&E slides were examined by three pathologists (IMP, FG and ALLB) all blinded to the clinical data. In cases with discordant results, a consensus was reached on a multi-head microscope. The following data were retrieved: histological grade: conventional SFT vs. dedifferentiated SFT; predominant tumor cell morphology: round cells, spindle cells, round and spindle cells, others (fat-forming, giant cells, epithelioid, pleomorphic cells); mitotic rate: ≥4/10HPF vs. <4/10HPF; necrosis: yes vs. no; cellularity: high vs. moderate vs. low; nuclear pleomorphism: yes vs. no; fibrosis: yes vs. no; myxoid pattern: yes vs. no.

### 4.3. Immunohistochemistry

IHC staining was carried out on 3–4 micron-thick FFPET from a single representative block for each primary tumor section. The primary antibodies, source, dilution and staining pattern criteria used are listed in Supplementary Table S1. The reactions were detected using the EnVision system (Dako, Glostrup, Denmark). Staining intensity was graded as negative, weak, moderate or strongly positive. The extent of positive IHC reaction was scored, as previously described [42]. All sections were evaluated independently and read in a blind manner by three pathologists (IM, FG and ALLB). Discordant cases were evaluated at a multi-head microscope to achieve consensus. Standard positive and negative controls were used throughout. The scores by all observers were recorded, and in cases of disagreement, the score was determined by consensus.

### 4.4. DNA/RNA Isolation, PCR, RT-PCR and Sequencing DNA

Genomic DNA was extracted from formalin-fixed, paraffin-embedded (FFPE) samples using a QIAamp DNA FFPE Tissue Kit (Qiacube automated system, Qiagen, Hilden, Germany) according to the manufacturer’s instructions. DNA quantification was performed using the Nanodrop One (Thermo Scientific, Waltham, MA, USA). PCRs for the TERT promoter and exons 5, 6, 7 and 8 for TP53 were performed in the Proflex PCR System (Applied Biosystems. Waltham, MA, USA). Sequencing reactions were carried out using BigDye v1.1 (Applied Biosystems) and analyzed on a SeqStudio Genetic Analyzer (Applied Biosystems). All sequencing analyses were read on both strands in order to exclude pre-analytical and analytical errors.

### 4.5. RNA

Total RNA was extracted from formalin-fixed, paraffin-embedded (FFPE) samples using a RNeasy FFPE Kit (Qiacube automated system, Qiagen, Hilden, Germany). We performed PCRs to detect NAB2/STAT6 gene fusions. Primers used are listed in Supplementary Table S2. Sequencing reactions were carried out using BigDye v1.1 (Applied Biosystems) and analyzed in a SeqStudio Genetic Analyzer (Applied Biosystems). All sequencing analyses were read on both strands in order to exclude pre-analytical and analytical errors. Non-informative cases were defined as those with no RT-PCR results.

### 4.6. Risk of Recurrence/Metastasis

The risk of recurrence/metastasis was calculated using the Demicco scoring system [1,2]. The criteria for the Demicco et al. scoring system are presented in Table 6.

## Figures and Tables

**Table 1:** Solitary fibrous tumor clinical data (N = 97).

Variables	N	%
Age		
<55	52	53.6
≥55	45	46.4
Gender		
Male	46	47.4
Female	51	52.6
Tumor location		
Limbs	6	6.2
Not limbs	91	93.8
Tumor location		
Soft tissue and/or subcutaneous tissues	64	66
Pleural	19	19.6
Visceral	12	12.3
Meningeal	2	2.1
Tumor size		
<5 cm	38	39.1
5–<10 cm	36	37.1
10–<15 cm	12	12.4
≥15 cm	11	11.4
Location		
Deep	90	92.8
Superficial	7	7.2
Surgery		
Yes	94	97
No	3	3
Radiotherapy		
Yes	4	4
No	93	96
Chemotherapy		
Yes	7	7.2
No	90	92.8

**Table 2:** Solitary fibrous tumor. Follow-up data (N = 97). Median follow-up (90 months).

Variables	N	%
Recurrence/Relapse		
Yes	9	9.3
No	88	90.7
Metastasis		
Yes	11	11.3
No	86	88.7
Recurrence/Relapse and metastasis		
Yes	4	4.1
No	93	95.9
Metastasis		
Lung	9	81.8
Others	2	18.2
Clinical outcome and current status		
Died of disease	10	10.3
Alive with disease	5	5.3
Alive, free of disease	74	76.2
Lost to follow-up	8	8.2

**Table 3:** Solitary fibrous tumor. Histological findings clinical (N = 97).

Variables	N	%
Histological pattern		
Conventional, no dedifferentiation	94	97
Dedifferentiated tumor	3	3
Predominant histological pattern		
round cells	3	3
spindle cells, patternless	58	59.9
spindle and round cells	16	16.5
others (giant-cells, fat-forming, epithelioid, pleomorphic)	20	20.6
Mitosis		
≥4/HPF (high power fields)	13	13.4
<4/HPF	84	86.6
Necrosis		
Yes	16	16.5
No	81	83.5
Cellularity		
High	37	38.1
Moderate	38	39.1
Low	22	22.8
Nuclear pleomorphism		
Yes	20	20.6
No	77	79.4
Myxoid pattern		
Yes	44	45.4
No	53	54.6

**Table 4:** Solitary fibrous tumor. Immunohistochemistry (N = 97).

Variables	N	%
STAT6		
Positive	97	100
Negative	0	0
Ki-67 index		
≥10	14	14.4
<10	83	85.6
CD99		
Positive	85	57.1
Negative	12	42.9
CD34		
Positive	92	92.9
Negative	5	7.1
Bcl2		
Positive	85	67.9
Negative	12	32.1
Synaptophysin Chromogranin-A, INSM1 (≥1 positive)		
Positive	25	25.7
Negative	72	74.3
Desmin, SMA, Myogenin (≥1 positive)		
Positive	4	14.3
Negative	24	85.7
Epithelial markers (CK, EMA)		
Positive	3	3
Negative	94	97
APAF-1		
Positive	84	87
Negative	13	13

**Table 5:** Solitary fibrous tumor, molecular results (N = 97).

Variables	N
*NAB2/STAT6*	
Positive *NAB2-exon 4/STAT6-exon 2*	35
Positive *NAB2-exon 6/STAT6-exon 16/17*	15
Non informative	47
*p53*	
Mutation	15 (17%)
WT (wild type)	71
Non informative	11
*HTER*	
Mutation	35 (40.6%)
WT	51
Non informative	11
*p53* and *HTER* (mutations)	
Yes	8 (9.3%)
No	78
Non informative	11

**Table 6:** Solitary fibrous tumor. Risk stratification system results and Demicco et al. system (N = 97).

Risk Stratitication Assigned by Demicco et al. System	N	%
High-risk SFT (solitary fibrous tumor)	6	6.1
Intermediat-risk SFT	11	11.3
Low-risk SFT	80	82.4
Total	97	100
**Demicco et al system, variables**		**scores**
Age	<55	0
	≥55	1
Tumor size (cm)	<5	0
	5 to <10	1
	10 to <15	2
	≥15	3
Mitosis (×10 high power fields/HPF)	0	0
	1–3	1
	≥4	2
Necrosis	<10	0
	≥10	1
Final score	Low	0–3
	Intermediate	4–5
	High	6–7

**Table 7:** High-risk solitary fibrous tumor (N = 6).

Cases	Mitosis ≥ 4	Necrosis	Ki-67 ≥ 10	*HTER*	*p53*	Metastasis and/or Recurrence	Outcome
1				WT	mutation		DOD
2				WT	WT		AWD
3				mutation	mutation		DOD
4				mutation	mutation		DOD
5				mutation	mutation		DOD
6				mutation	WT		AWD

Red: yes, WT: wild type, DOD: died of disease, AWD: alive with disease.

**Table 8:** Intermediate-risk solitary fibrous tumor (N = 11).

Cases	Mitosis ≥ 4	Necrosis	Ki-67 ≥ 10	*HTER*	*p53*	Metastasis and/or Recurrence	Outcome
1		no	10	mutation	WT		DOD
2	no	no	<5	mutation	WT		DOD
3	no	no	<5	mutation	WT		LFU
4	no		5	mutation	WT		LFU
5		no	10	mutation	WT		DOD
6	no		<5	mutation	WT		AWD
7	no		<5	WT	WT	no	NED
8			5	mutation	WT		DOD
9			10	WT	mutation	no	DOC
10		no	10	mutation	WT	no	NED
11	no	no	<5	WT	mutation	no	NED

Red: yes, DOD: died of disease, WT: wild type, AWD: alive with disease, NED: no evidence of disease, LFU: lost to follow-up, DOC: death from other causes.

**Table 9:** Low-risk solitary fibrous tumor with poor evolution (N = 4).

Cases	Age	Tumor Size	Mitosis ≥ 4	Necrosis	Desd	Ki67%	HTER	p53	Metastasis and/or Recurrence	Outcome
1	25	10	no	no	yes	25	WT	WT		AWD
2	51	24	no	no	no	5	WT	WT		DOD
3	42	4	17	no	no	70	WT	WT		DOD
4	52	8	5	no	no	5	WT	WT		AWD

Red: yes and/or adverse factor, DOD: died of disease, AWD: alive with disease.

## Data Availability

Data is contained within the article or Supplementary Material. In addition, any additional data presented in this study are available and would be requested to the corresponding author.

## Supplementary Materials

The following are available online at https://www.mdpi.com/article/10.3390/ijms22179423/s1.
