# Supplementary material for: Solitary Fibrous Tumor: Integration of Clinical, Morphologic, Immunohistochemical and Molecular Findings in Risk Stratification and Classification May Better Predict Patient outcome
Source: Int J Mol Sci. 2021 Aug 30;22(17):9423. doi: 10.3390/ijms22179423 (PMC8430583; doi:10.3390/ijms22179423)
Supplement: Supplementary file 1 [file ijms-22-09423-s001.zip › ijms-1316795-supplementary.pdf]

Supplementary Table S1. Immunohistochemistry analysis. Antibodies, sources, clone, dilutions, pretreatments and staining patterns.

Antibodies	Source	Clone	Dilution	Pretreatment	Staining pattern
CD99	DAKO IR057	12E7	prediluted	PTLINK high pH	membranous
CD34	DAKO IR632	QBEnd	prediluted	PTLINK low pH	cytoplasmic
BCL2	DAKO IR614	123	prediluted	PTLIMK high pH	cytoplasmic
STAT6	ABCAM AB32520	YE361	1/1000	AUTOCLAVE	nuclear
Ki67	DAKO IR626	MIB-1	prediluted	PTLINK low pH	nuclear
INSM1	SANTA CRUZ BIOTHECNOLOGY	A-8	1/200	PTLINK high pH	nuclear
Synaptophysin	DAKO IR660	DAK-SYNAP	prediluted	PTLINK high pH	cytoplasmic
Chromogranin	DAKO M0869	DAK-A3	1/50	PTLINK high pH	cytoplasmic
SMA	DAKO	1A4	prediluted	PTLINK high pH	cytoplasmic
Desmin	DAKO IR606	D33	prediluted	PTLINK high pH	cytoplasmic
Myogenin	DAKO IR067	F5D	prediluted	PTLINK high pH	nuclear
CK(AE1/AE3)	DAKO IR053	AE1/AE3	prediluted	PTLINK low pH	cytoplasmic/membranous
EMA	DAKO	Clon E29	Ready to use	PTLINK low pH	cytoplasmic/membranous
APAF-1	ABCAM	EPR21112-102	prediluted	PTLINK low pH	nuclear

Supplementary Table S2. Primers to molecular analysis (*NAB2/STAT6*, *TP53* and *TERT)*

	**Primers**	**Sequencing 5´ to 3´**	**PCR fusion products**	**Observations**
Gene fusion	*NAB2-2/STAT6-2RF*	AGG AGA TCC GCA AAT ACA GC	*NAB2 exon2 – STAT6 exon 2*	
Transcript NAB2-201	*NAB2-2/STAT6-2.RR*	GGC ATC TTG GAG ACC AGA CC		
**Transcript *STAT6-201***	*NAB2-2/STAT6-6RF*	CAT CTA TGG CCG TTT CGA CTC	*NAB2 exon2 – STAT6 exon 6*	
	NAB2-2/STAT6-6.RR	TCG CTC TAG CTC TCC AGT GG		
	NAB2-4/STAT6-2RF	GCT TCA CCC TGA AGA ACT GGG A	*NAB2 exon4 – STAT6 exon 2*	
	NAB2-4/STAT6-2.RR	AGT CGA CAT AGA GCC GCT GC		
	NAB2-4/STAT6-3RF	CCT AGT TCT GCA TGA GG AC	*NAB2 exon4 – STAT6 exon 3*	
	NAB2-4/STAT6-3.RR	TAG GGC ACT AGC CAA GTT GCA		
	NAB2-6/STAT6-16-17RF	CAC TGA TGG ACG AGG GGC TG	*NAB2 exon6 – STAT6 exon 16*	
	NAB2-6/STAT6-16.RR	AGC AAG ATC CCG GAT TCG GTC		
	NAB2-6/STAT6-17.RR	CCA TGG TAG GCA TCT GGA GC	*NAB2 exon6 – STAT6 exon 17*	
	NAB2-6/STAT6-18.RF	GCT ATG GAG CCG ACA CAT CC	*NAB2 exon6 – STAT6 exon 18*	
	NAB2-6/STAT6-18.RR	GGA ATC AGG GGC CAT TCC AA		
	NAB2-7 – STAT6-2.RF	AAG CCA CCT CTC GCA GAG TTC GA	*NAB2 exon7 – STAT6 exon 2*	
	NAB2-4 – STAT6-2.RR	AGT CGA CAT AGA GCC GCT GC		
TERT promotor	*PROM.TERT.DF*	CAGCGCTGCCTGAAACTCG		*TERT promotor gene*
	PROM.TERT.DR	CCACGTGGCGGAGGGACT		
TP53	*P53 5DF*	CTGACTTTCAACTCTGTCTC		*Exon 5*
Transcript EWSR1-208	*P53 5DR*	CAACCAGCCCTGTCGTCTCT		
	P53 6DF	CTCTGATTCCTCACTGATTG		*Exon 6*
	P53 6DR	GGCCACTGACAACCACCCTTAACC		
	P53 7DF	CTCATCTTGGGCCTGTGTTA		*Exon 7*
	P53 7DR	AGTGTGCAGGGTGGCAAGTG		
	P53 8DF	ACCTGATTTCCTTACTGCCTCTTGC		*Exon 8*
	P53 8DR	GTCCTGCTTGCTTACCTCGCTTAGT		
